# Berberine alleviates atherosclerosis by modulating autophagy and inflammation through the RAGE-NF-κB pathway

**DOI:** 10.3389/fphar.2025.1540835

**Published:** 2025-03-31

**Authors:** Peng Zhang, Meiying Jin, Lei Zhang, Yanjun Cui, Xiaokang Dong, Jie Yang, Jiayu Zhang, Haopeng Wu

**Affiliations:** ^1^ College of traditional Chinese medicine, Binzhou Medical University, Yantai, China; ^2^ Department of Geriatrics, Yantai Affiliated Hospital of Binzhou Medical College, Yantai, China; ^3^ Department of Cardiovascular, Affifiliated Hospital of Shandong University of Traditional Chinese Medicine, Jinan, China; ^4^ Department of Ultrasound, Affifiliated Hospital of Shandong University of Traditional Chinese Medicine, Jinan, China

**Keywords:** atherosclerosis, berberine, autophagy, RAGE-NF-κB signaling pathway, network pharmacology

## Abstract

**Introduction:**

Lipid accumulation and foam cell formation are significant features that expedite the progression of atherosclerosis (AS). Abnormal autophagy is a key factor in the development of AS. The importance of berberine (BBR) in AS has been well established. However, its exact role in regulating autophagy and alleviating atherosclerotic inflammation remains unclear.

**Purpose:**

This study was aimed at exploring the role and mechanism of BBR in alleviating AS by activating autophagy and alleviating inflammation.

**Study design:**

Network pharmacology predicts the potential mechanism of BBR in regulating AS and verifies this mechanism through *in vivo* and *in vitro* experiments, thereby providing new thinking for clinical treatment.

**Methods:**

The potential mechanism through which BBR regulates AS was predicted by network pharmacology. Total cholesterol (TC), triglyceride (TG), low-density lipoprotein cholesterol (LDL-C), and high-density lipoprotein (HDL-C) were measured by administering BBR (100 mg/kg) via the stomach. Hematoxylin and eosin (HE) and oil red O staining were used for histological analysis. Expression levels of the RAGE and p-NF-κB pathways and autophagy-associated proteins were evaluated by immunofluorescence. The ApoE^−/−^ mouse model was established with a high-fat diet (HFD) to verify the effect and mechanism of BBR *in vivo*.

**Results:**

Functional and pathway enrichment analysis demonstrated that BBR significantly modulated the inflammation-related signaling pathways of AS. Additionally, *in vivo* experiments indicated that BBR reduced aortic lipid deposition and reduced the atherosclerotic plaque area. BBR decreased the expression levels of RAGE, p-NF-κB, TNF-α, and P62 in the aorta, and upregulated the expression levels of IL-10, CD31, VEGF, LC3B, and Beclin1. Similar results were obtained in vitro experiments, further supporting the *in vivo* findings. Notably, NF-κΒ activator 1 attenuated the effect of BBR.

**Conclusion:**

In summary, BBR alleviated the disease progression of AS by regulating the expression of RAGE and p-NF-κB and activating autophagy.

## 1 Introduction

Atherosclerosis (AS) is a chronic inflammatory disease characterized by the abnormal accumulation of lipids, immune cells, and fibrous mediators in the subendothelial layer of the arteries (Tabaei and Tabaee, 2019). During the course of the disease, the deposition of low-density lipoprotein (ox-LDL) in the intima of the artery activates the endothelial cells to secrete adhesion factors, giving rise to the production of M2 macrophages and promoting inflammation (Koelwyn GJ, et al., 2018; Malekmohammad et al., 2019). The accumulation of foam cells leads to the formation of fibrous plaques and the generation of atherosclerotic plaques, thereby accelerating the progression of the disease (Basatemur et al., 2019; Libby, 2012). Autophagy is a process driven by a double-membrane vesicle that involves the transportation of cytoplasmic material to lysosomes for degradation and recycling, thus contributing to the metabolism of proteins, glucose, and lipids (Yang and Kilonsky, 2010; Nakatogawa et al., 2009; Boya et al., 2013). Increasing evidence indicates that dysregulated autophagy is associated with the progression of atherosclerosis (Nussenzweig et al., 2015; Gatica et al., 2015). In the early stages of atherosclerotic lesion formation, autophagy inhibits apoptosis of vascular endothelial cells, reduces plaque size, stabilizes plaque phenotype, and slows plaque progression (Shan et al., 2021). In advanced stages of AS, however, excessive autophagy can lead to vascular cell death, reduced collagen synthesis, weakening of fibrous caps, and plaque rupture (Li H. et al., 2016). Therefore, understanding the underlying mechanisms of AS is crucial. Natural metabolites from medicinal plants, which have gained attention for their anti-inflammatory effects and relatively fewer side effects, have emerged as pharmacological candidates for treating AS (Park et al., 2019). Berberine (BBR), an isoquinoline alkaloid isolated from *Coptis chinoides* and other plants in the *Berberis* genus, exhibits various pharmacological properties, such as antioxidant, anti-inflammatory, and autophagy-regulatory properties, as well as a capacity to reduce mitochondrial damage and cell death (Eftekhari, et al., 2020; Hashemzaei and Rezaee, 2021; Rui et al., 2022). Recent studies have emphasized its role in inhibiting tumor growth (Katiyar et al., 2009), as well as regulating lipid metabolism (Kong et al., 2004). Moreover, BBR may inhibit AS formation and enhance the stability of atherosclerotic plaques in ApoE^−/−^ mice by activating ERK/JNK, AMPK, and PPARγ (Wang et al., 2011; Li L. et al., 2016; Chen et al., 2014; Zhu et al., 2018). As research advances, BBR has shown a protective role in cardiovascular diseases by modulating autophagy (Mohammadinejad et al., 2019). Huang et al. discovered that BBR alleviated myocardial ischemia/reperfusion injury by inhibiting excessive autophagy of cardiomyocytes (Huang et al., 2015). Li et al. demonstrated that BBR alleviates stress-induced cardiac hypertrophy and dysfunction through the induction of autophagy, a process that exerts a beneficial effect in this context. (Li et al., 2014). Fan et al. also demonstrated that BBR alleviates ox-LDL-induced inflammatory factors in AS by upregulating autophagy (Fan et al., 2015). However, the exact mechanism by which BBR-mediated autophagy in AS alleviates inflammatory responses is largely unknown.

**FIGURE 1:**
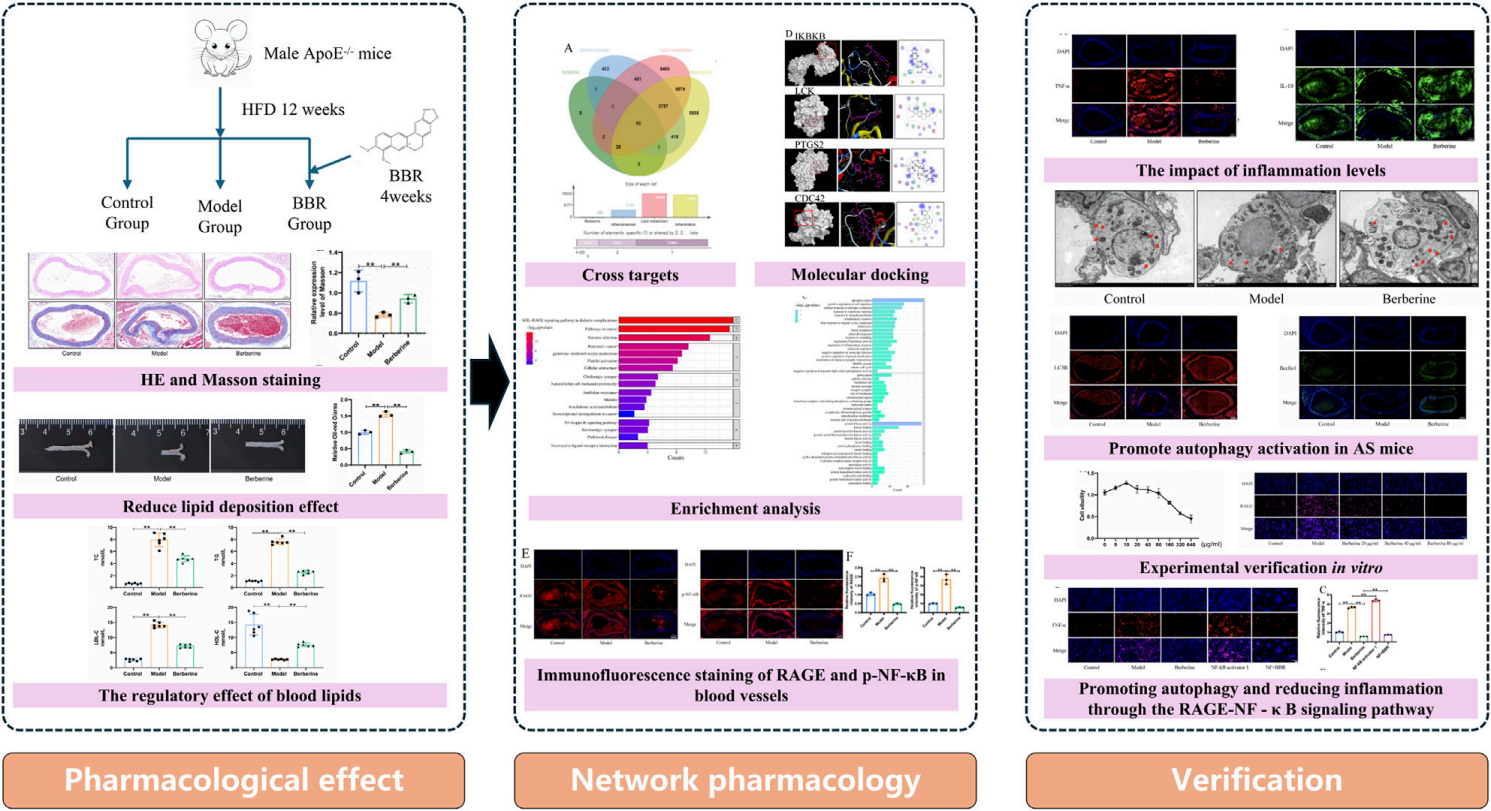
Experimental design diagram.

Network pharmacology is commonly used to predict drug targets by constructing a “disease-gene-target-drug” network (Hopkins, 2007; Di et al., 2021; Hopkins, 2008). It has been validated as a useful bioinformatics tool to collect potential targets of virtually all bioactive metabolites to treat disease and to discover core targets and signaling pathways (Li et al., 2021), representing an effective approach for multi-target drug treatment (Di et al., 2021). Molecular docking, a method used to predict ligand-receptor binding patterns and affinities based on ligand interaction patterns, further helps in understanding drug function and mechanism (Pinzi and Rastelli, 2019). This study predicted the potential targets and mechanisms of BBR in alleviating AS through network pharmacology and molecular docking and verified the effects and mechanisms of BBR on AS through *in vitro* and *in vivo* experiments (Figure 1).

## 2 Materials and methods

### 2.1 Materials

Berberine (2086-83-1) was acquired from Desite Biotechnology Co., LTD., (Chengdu, China); Triglyceride (TG) Content Assay Kit, Total Cholesterol (TC) Content Assay Kit, Low-Density Lipoprotein Cholesterol (LDL-C) Content Assay Kit, and High-Density Lipoprotein Cholesterol (HDL-C) Content Assay Kit were purchased from Nanjing Jiancheng Bioengineering Institute (Nanjing, China); NF-κΒ activator 1 was purchased from MedChemExpress (Shanghai, China); Antibodies against Beclin1, LC3B, P62, CD31, VEGF, TNF-α, and IL-10 were purchased from Proteintech (Wuhan, China); Antibodies against RAGE and p-NF-κB were purchased from Thermo Fisher.

### 2.2 Analysis of network pharmacology

Genes related to BBR and AS were obtained from six databases: (https://old.tcmsp-e.com/tcmsp.php), GeneCards (https://www.genecards.org/), PharmMapper (http://lilab-ecust.cn/pharmmapper/index.html), Swiss Target Prediction (http://swisstargetprediction.ch/), and STITCH (http://stitch.embl.de/). Targets were normalized and converted to gene names using the UniProt database (https://www.uniprot.org/), and duplicates were removed to compile the final list of BBR targets. To identify disease-related targets, “Atherosclerosis,” “Lipid metabolism,” and “Inflammation” were used as keywords in DisGeNET (https://www.disgenet.org/), GeneCards, and the Online Mendelian Inheritance in Man (OMIM) database (https://omim.org/). After removing duplicates, a Venn diagram was generated to visualize the intersections of drug- and disease-related targets. Cross-gene pathway analysis was conducted with Kyoto Encyclopedia of Genes and Genomes (KEGG) pathway enrichment, using the DAVID Bioinformatics Resources (https://david.ncifcrf.gov/).

### 2.3 Molecular docking verification

The three-dimensional (3D) structure of BBR (in MOL2 format) was acquired from the PubChem database (https://pubchem.ncbi.nlm.nih.gov/), while those of target genes, used as protein receptors, were obtained from the RCSB PDB database (http://www.rcsb.org/). Using PyMOL software, the target protein receptors were prepared by dehydration, followed by hydrogenation with AutoDockTools 1.5.6. Molecular docking and calculation of docking energy values were performed with AutoDock Vina, where an affinity value of ≤−5.0 kcal/mol was considered to indicate strong interaction between the receptor and ligand (Liu et al., 2022). PyMOL was used to visualize the 3D predicted binding sites, while LigPlus was used to visualize the corresponding 2D predicted binding sites.

### 2.4 Animals and cells

Male ApoE^−/−^ mice were purchased from Beijing SPF Biotechnology Co., LTD., and housed at the Peking University Health Science Center. All animal experiments were conducted following the guidelines of the Laboratory Animal Management Committee of Peking University Health Science Center. The mice were subjected to a 12 h light/12 h dark cycle under standard housing conditions (room temperature: 22°C ± 2°C, humidity: 55% ± 10%). Male ApoE−/− mice were fed a high-fat diet (Research Diets, Inc., D12109C) for 12 weeks and randomly divided into three groups. The ApoE^−/−^ + BBR group received 100 mg/kg of BBR via daily gavage for a period of 4 weeks (Wu et al., 2020). The Human Umbilical Vein Endothelial Cells (HUVEC) line was purchased from Cyagen (Suzhou, China). HUVEC cells were stimulated with 50 μg/mL ox-LDL for 48 h. Oil red O staining revealed that the cell volume expanded and became round, short-spindle-shaped or irregular, with numerous red or dark red round lipid droplets in the cytoplasm, and the cell foam was remarkable. Subsequently, Berberine at concentrations of 20 μg/mL, 40 μg/mL, and 80 μg/mL was administered for 24 h.

### 2.5 Histological examination

The mice were fasted for 8 h before euthanasia. After administering abdominal anesthesia, blood samples were collected from the inferior vena cava, and the perivascular adipose tissue (PVAT) and aorta were isolated on ice. Euthanasia was performed under excessive anesthesia (80 mg/kg pentobarbital sodium, intraperitoneally). The aortic root and valve were sectioned using a microtome, and 10 μm frozen sections were prepared for oil red O and Masson staining. The aorta and PVAT were stained with hematoxylin and eosin (HE) and examined under a microscope (Carl Zeiss, Germany). Image analysis was performed using ImageJ software (Version 1.52a, NIH, Bethesda, MD, United States).

### 2.6 Oil red O staining

Lipid deposition in the aorta was assessed using oil red O staining. The aorta was quickly removed under anesthesia, washed with phosphate-buffered saline (PBS) at 4°C, and fixed overnight with 4% paraformaldehyde. The aorta was then cut lengthwise to expose the intima surface or at the entrance to the aorta. After 1 h of 0.5% oil red O staining, the aorta was differentiated in 60% propylene glycol solution for 5 min. The stained aorta was photographed under consistent lighting conditions and analyzed using ImageJ software.

### 2.7 Transmission electron microscopy (TEM)

Aortic tissue samples were fixed with 2.5% glutaraldehyde for 2 h at 4°C, then stored overnight at 4°C. Samples were dehydrated at room temperature, resin permeated, and embedded. Resin-embedded models and samples were polymerized in an oven at 65°C for more than 48 h. The resin blocks were then removed from the embedded model and sectioned into 60–80 nm slices using an ultra-thin microtome (No. EM UC7 Ultramicrotome, Leica Microsystems, Wetzlar, Germany). The sections were mounted on a copper grid, stained with uranyl acetate saturated alcohol solution (2%), and kept in the dark for 8 min, followed by three washes with 70% ethanol and ultrapure water. Next, the samples were stained with 2.6% lead citrate for 8 min and washed three times with ultrapure water. After drying, the copper grid was observed under a transmission electron microscope and images were taken.

### 2.8 Analysis of cell viability

Cell viability was measured using the CCK-8 kit (Biosharp, Shanghai, China). Cells were treated with varying concentrations of BBR to establish the cell model. After 24 h of incubation, 10 µL of CCK-8 reagent was added to each well, followed by an additional 4-h incubation at 37°C. Absorbance was then measured at a detection wavelength of 450 nm, with a reference wavelength of 630 nm. Cell viability was calculated based on the absorbance readings.

### 2.9 Immunofluorescence assay

Aortic tissue sections or cell slides were incubated with primary antibodies (1:200) against RAGE, p-NF-κB, TNF-α, IL-10, CD31, VEGF, LC3B, Beclin1, and P62 at 4°C for 1 h. Subsequently, the sections were incubated with a fluorescent secondary antibody (Thermo, 1:1,000) for 1 h. The nuclei were stained with DAPI, and fluorescence images were captured using a fluorescence scanning microscope for analysis.

### 2.10 ELISA detection method

Vascular tissue of uniform size was homogenized in normal saline and centrifuged at 3,000 rpm for 10 min at 4°C. The supernatant was collected, and the protein concentration was measured using BCA assay (P0010). The samples were then adjusted to the same protein concentration before performing the ELISA assay to measure the levels of interleukin - 6 (IL - 6, MB - 2899A) and IL - 10 (MB - 2912A) following the ELISA protocol, and the absorbance was measured using a microplate reader.

### 2.11 The relative expression levels of CD31 and VEGF as detected by Q-PCR

RNA was extracted from blood vessel tissues of uniform size and reverse-transcribed into cDNA, which was then stored at −20°C. SYBRTM PreMix-Ex TagTM (Takara, Dalian, China) was used to detect the relative expression levels of CD31 and VEGF mRNA in a real-time PCR system. The primer sequences are in Table1.

**TABLE 1:** The primer sequences.

mRNA	Forward Primer (5′-3′)	Reverse Primer (5′-3′)
CD31	CCA​AAG​CCA​GTA​GCA​TCA​TGG​TC	GGA​TGG​TGA​AGT​TGG​CTA​CAG​G
VEGF	CTG​CTG​TAA​CGA​TGA​AGC​CCT​G	GCT​GTA​GGA​AGC​TCA​TCT​CTC​C
GAPDH	CAT​CAC​TGC​CAC​CCA​GAA​GAC​TG	ATG​CCA​GTG​AGC​TTC​CCG​TTC​AG

### 2.12 Statistical analysis

All data were statistically analyzed and visualized using GraphPad Prism 9.0. Data are expressed as mean ± standard error of the mean (SEM) and were compared using one-way analysis of variance (ANOVA) followed by a *post hoc* Tukey test. Statistical significance was set at *P* < 0.05.

## 3 Results

### 3.1 BBR mitigated the deposition of aortic plaques in AS

**FIGURE 2:**
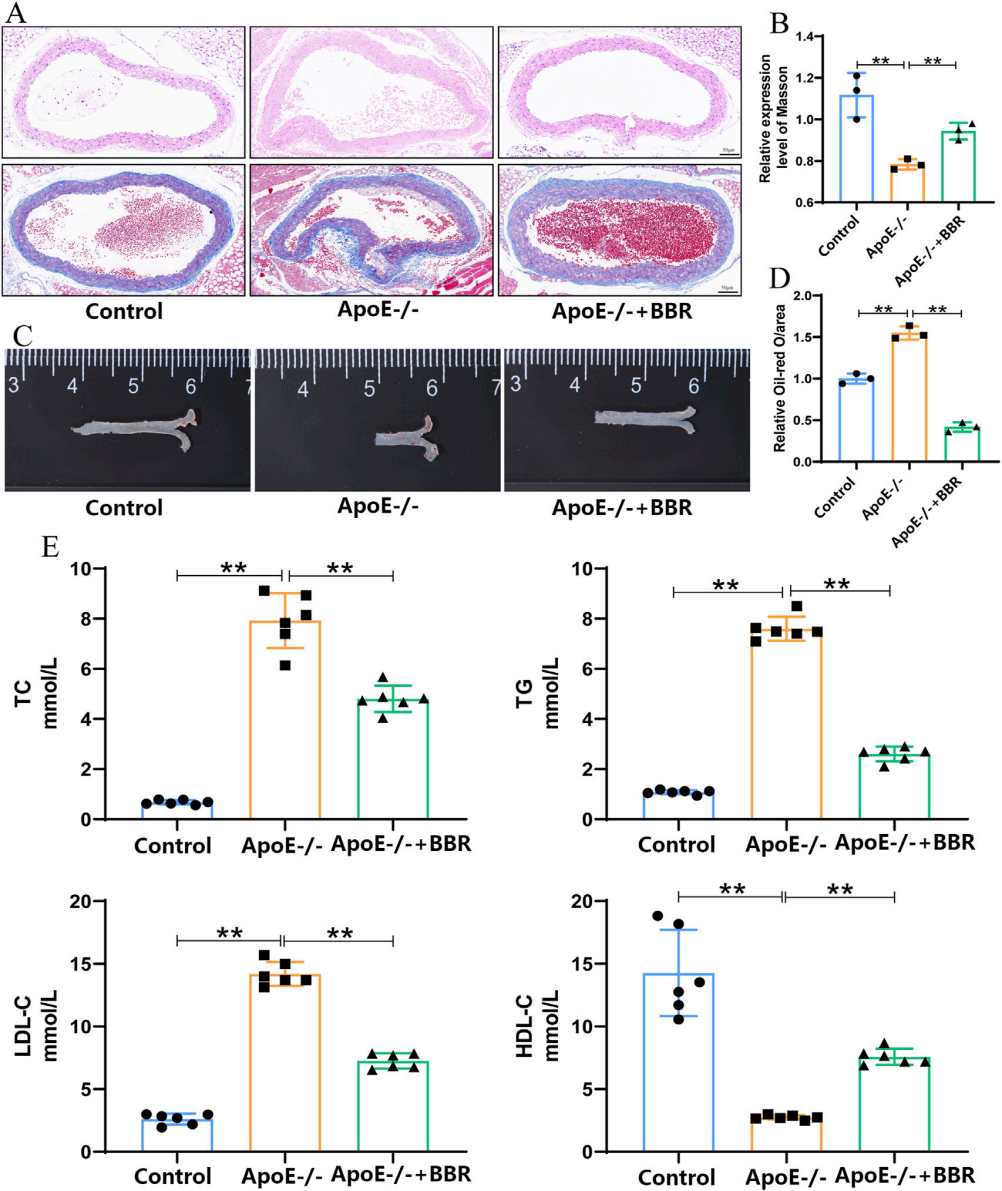
BBR mitigated the deposition of aortic plaques in AS. **(A)** HE and Masson staining of mouse aortic root (scale = 50 μm). **(B)** Quantitative analysis of mouse aorta following Masson staining, where data are presented as the mean ± standard deviation (n = 3). **(C)** Representative image of aorta following oil red O staining. **(D)** Quantitative analysis of lipid content, where data are presented as mean ± standard deviation (n = 3). **(E)** Effects of BBR on serum levels of TC, TG, LDL-C, and HDL-C in mice after treatment (n = 6 independent experiments). **P < 0.01.

To investigate the formation of lipid deposits in atherosclerotic plaques, HE staining was employed to observe the cross-section of the aortic root. The results of HE staining revealed that, in contrast to the control group, HFD intervention exacerbated aortic damage, facilitated plaque formation at the aortic root, and gave rise to the development of necrotic nuclei within the plaques, thereby causing a significant increase in lipid deposition. Nevertheless, treatment with BBR markedly alleviated these pathological features induced by HFD (Figure 2A). Masson staining of the aorta demonstrated that the fibrous cap thickness and collagen content increased in the ApoE^−/−^ + BBR group compared to the ApoE^−/−^ group (Figures 2A, B). As shown in Figure 2C, after prolonged exposure to HFD, the aortas of ApoE^−/−^ group exhibited extensive lipid deposition and severe atherosclerotic lesions. The area of atherosclerotic plaques at the aortic root was significantly larger than that in the aorta of normal mice and displayed a clumpy distribution. Following the administration of BBR, the distribution of lipid deposits in the aorta of the ApoE^−/−^ + BBR group was significantly reduced, presenting a punctate distribution (Figures 2C, D). Additionally, the results of the blood lipid test indicated that BBR significantly decreased the levels of TC, TG, and LDL-C induced by the HFD while increasing the levels of HDL-C (Figure 2E). These findings suggest that BBR can reduce aortic lipid deposition, decrease the area of atherosclerotic plaques, and effectively delay the progression of atherosclerosis.

### 3.2 Network pharmacology and molecular docking of BBR

**FIGURE 3:**
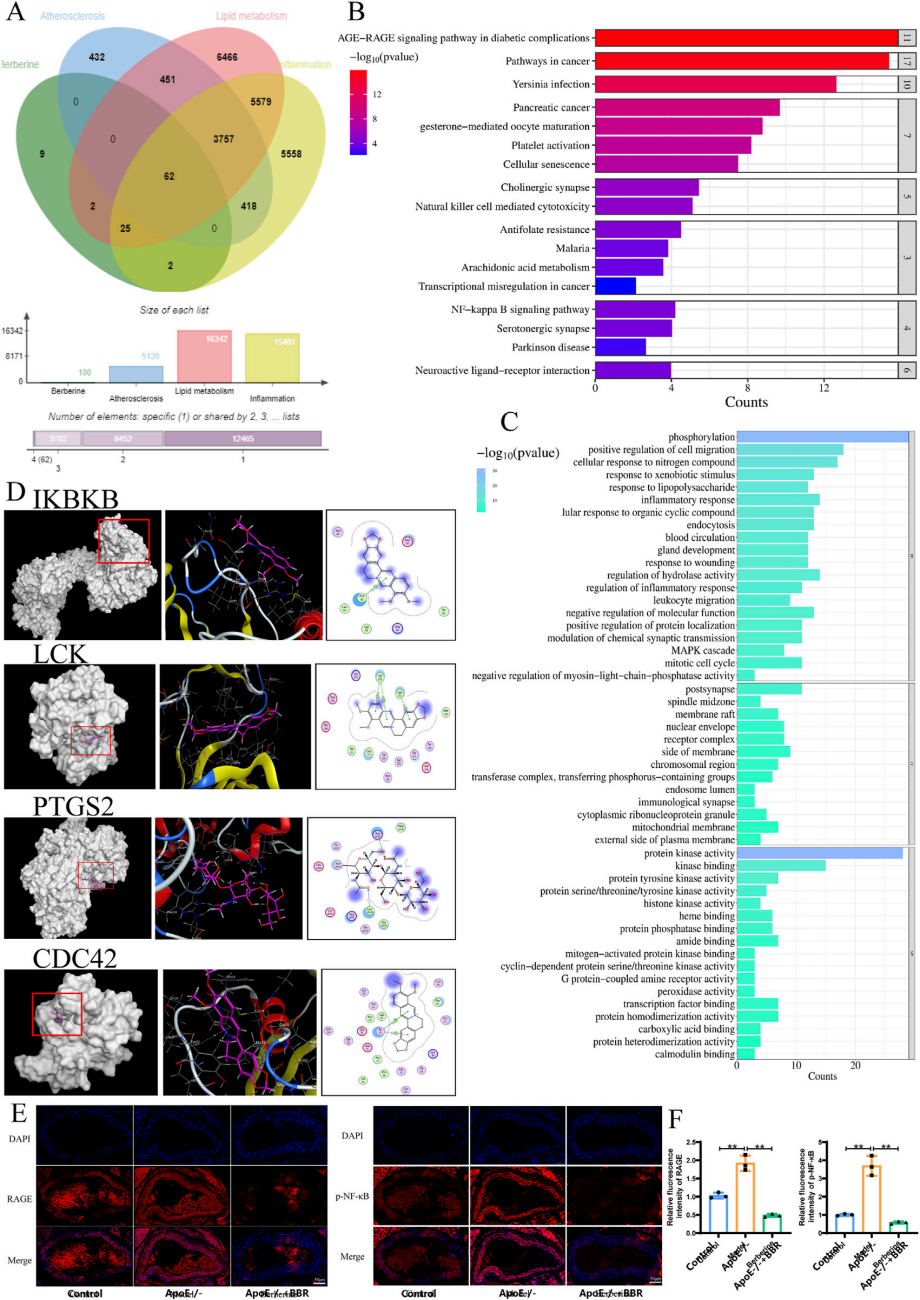
Network pharmacology and molecular docking of BBR. **(A)** Cross targets associated with AS, lipid metabolism, inflammation, and BBR. **(B)** KEGG pathway enrichment analysis of 62 cross targets. **(C)** GO pathway enrichment analysis of 62 cross targets. **(D)** 3D and 2D visualizations of BBR docking with IKBKB, LCK, PTGS2, and CDC42. **(E)** Immunofluorescence staining of RAGE and p-NF-κB in blood vessels (scale: 50 μm). **(F)** Quantitative immunofluorescence analysis of RAGE and p-NF-κB in blood vessels (n = 3).

To predict the potential mechanism of BBR, this study employed a network pharmacological approach. Using the keywords “Atherosclerosis,” “Lipid metabolism,” “Inflammation,” and “Berberine”, the study identified 100, 5,120, 16,342, and 15,401 related targets, respectively, from the RCSB PDB database. Among these, 62 targets were co-associated with both the disease and the drug (Figure 3A). Since the agreement between pathways predicted by bioinformatics methods and microarray confirmation was higher than that of predicted genes (Fang et al., 2017), we focused on enrichment pathways rather than specific genes. The KEGG that showed significant enrichment among the 62 targets included the AGE-RAGE signaling pathway in diabetic complications, the NF-κB signaling pathway, and neuroactive ligand-receptor interaction, indicating that the effect of BBR on AS is closely related to inflammation (Figure 3B). The enriched pathways in the GO analysis also emphasized inflammation and lipid metabolism processes (Figure 3C).

Schrodinger and PyMOL software were used for molecular docking to further verify the binding ability and interaction patterns of BBR with core target proteins related to lipid metabolism and the inflammatory response in AS. Key target proteins predicted by network pharmacology, associated with the AGE-RAGE and NF-κB signaling pathways, were used as receptors, while BBR, the key active metabolite, served as the ligand. CDC42, CDK4, F3, ICAM1, IKBKB, JAK2, LCK, MAPK10, MAPK14, PIM1, PTGS2, and RAC1 were selected as docking receptors. The molecular docking results obtained using AutoDock are summarized in Supplementary Table 1. The stability of the receptor-ligand binding was determined by the binding energy, where a binding energy of <−5.0 kcal/mol indicated good binding affinity to the proteins (Chen et al., 2021). The conformations of major hub targets and BBR are shown in Figure 3D and Supplementary Figure 1. BBR exhibited a strong binding affinity with AGE-RAGE pathway-related target proteins CDK4, MAPK14, PIM1, MAPK10, and RAC1, as well as with NF-κB pathway-related target proteins IKBKB, LCK, and PTGS2. The effects of BBR on the RAGE-NF-κB signaling pathway, atherosclerotic inflammatory response, and vascular endothelial cells were observed by immunofluorescence. As shown in Figures 3E, F, compared to the control group, the expressions of RAGE and p-NF-κB in the blood vessels of the ApoE^−/−^ group were significantly upregulated, and the expressions of RAGE and p-NF-κB were significantly downregulated after BBR treatment.

### 3.3 The impact of BBR on the inflammation level of AS

**FIGURE 4:**
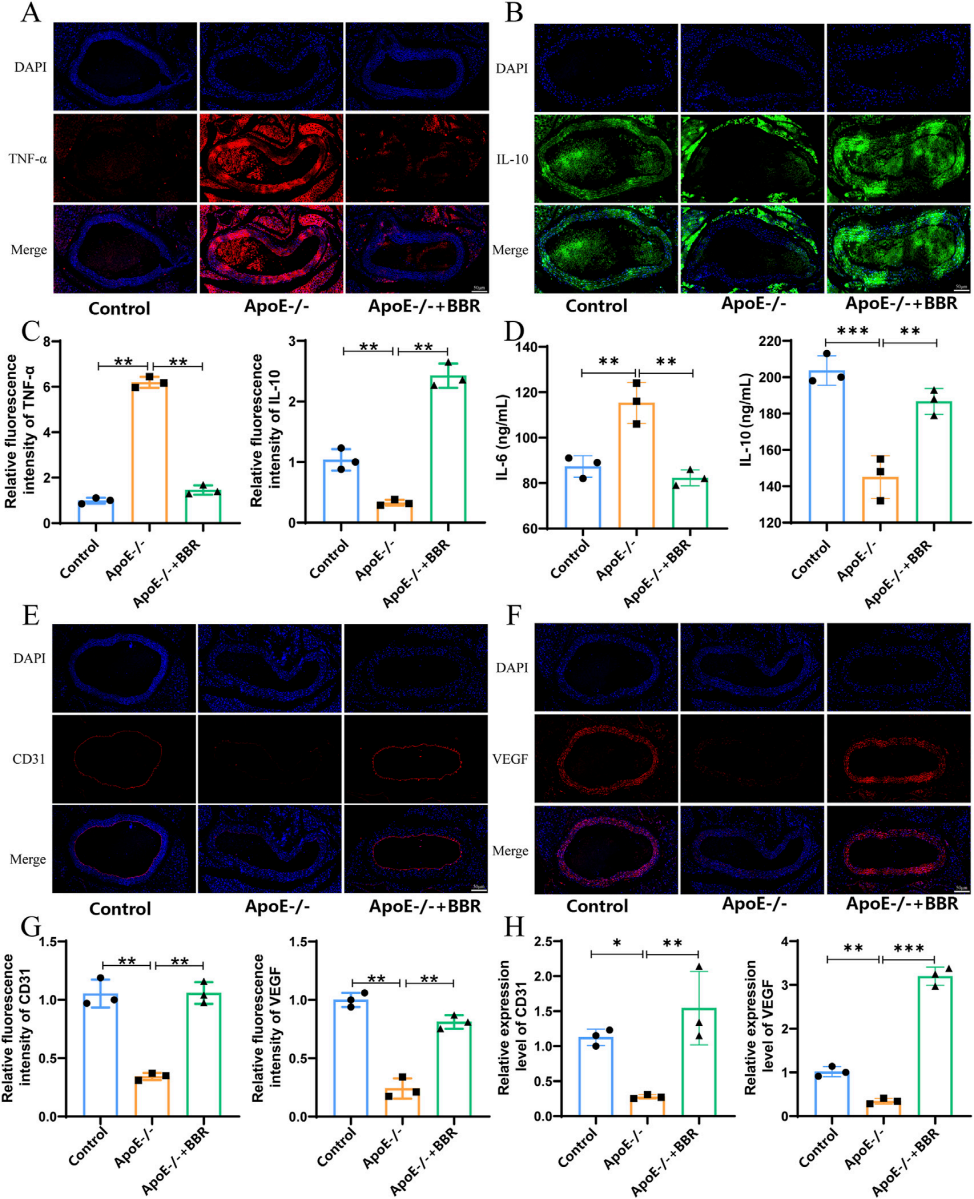
The impact of BBR on the inflammation level of AS. **(A)** Immunofluorescence staining of vascular TNF-α (scale: 50 μm). **(B)** Immunofluorescence staining of vascular IL-4 (scale: 50 μm). **(C)** Quantitative analysis of blood vessel TNF-α and IL-4 by immunofluorescence (n = 3). **(D)** Blood vessel IL-6 and IL-10 levels were measured by ELISA (n = 3). **(E)** Immunofluorescence staining of vascular CD31 (scale: 50 μm). **(F)** Immunofluorescence staining of vascular VEGF (scale: 50 μm). **(G)** Quantitative immunofluorescence analysis of CD31 and VEGF in blood vessels (n = 3). **(H)** qPCR was used to detect the relative expression levels of CD31 and VEGF mRNA in blood vessels (n = 3).

To further understand the effect of BBR on atherosclerotic inflammation, we established a mouse model of AS for *in vivo* validation. The effects of BBR on the RAGE-NF-κB signaling pathway, atherosclerotic inflammatory response, and vascular endothelial cells were assessed using immunofluorescence. As shown in Figures 4A–D, the expression of some inflammation-related protein markers such as pro-inflammatory factor TNF-α was upregulated in the ApoE^−/−^ group, and the expression of anti-inflammatory factor IL-10 was downregulated in the ApoE^−/−^ group, while BBR treatment significantly reversed these changes. ELISA results indicated that after BBR treatment, the tissue levels of IL-6 were significantly decreased, while the levels of IL-4 were significantly increased, consistent with the immunofluorescence findings (Figure 4D). Endothelial cell marker (CD31) and vascular endothelial growth factor (VEGF) were significantly expressed in aortic endothelial cells of mice treated with BBR, as demonstrated by immunofluorescence and qPCR analysis (Figures 4E–H), suggesting that BBR may maintain the integrity of vascular endothelial cells. These findings confirm that BBR significantly inhibits proteins associated with the RAGE-NF-κB signaling pathway, alleviates inflammatory responses, and aids in the repair of damaged vascular endothelial cells.

### 3.4 BBR treatment is capable of promoting the activation of autophagy in AS mice

**FIGURE 5:**
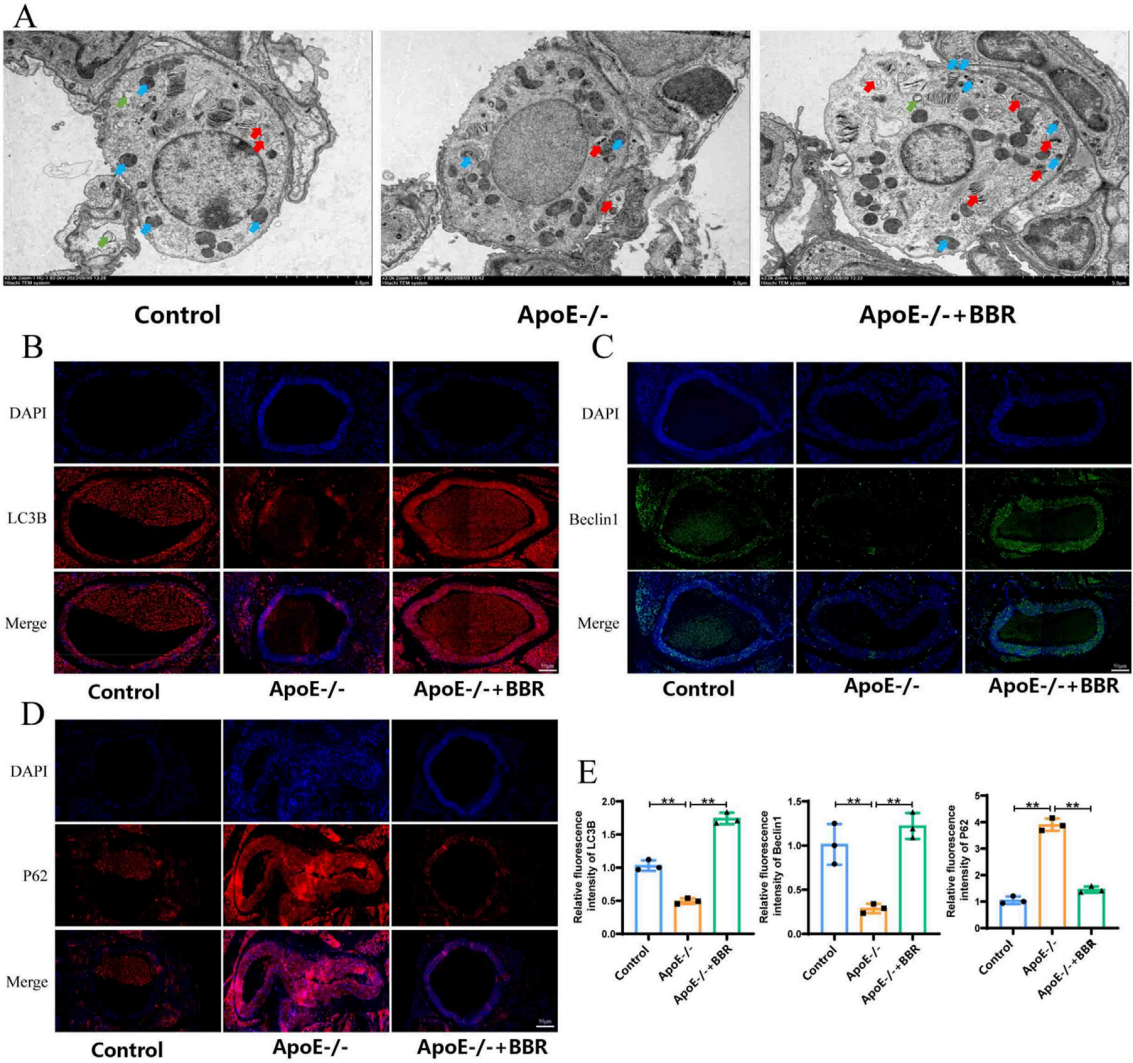
BBR treatment is capable of promoting the activation of autophagy in AS mice. **(A)** Autophagy in blood vessels was observed by TEM (ma gnified × 2,500, scale = 2 μm; green arrows: autophagic vacuoles, blue arrows: autophagosomes, red arrows: autolysosomes). **(B)** Immunofluorescence staining of LC3B in tissue samples (scale: 50 μm). **(C)** Immunofluorescence staining of Beclin-1 in tissue samples (scale: 50 μm). **(D)** Immunofluorescence staining of P62 in tissue samples (scale: 50 μm). **(E)** Immunofluorescence quantitative analysis of LC3B, Beclin-1, and P62 in tissue samples (n = 3).

Autophagy plays a key role in delaying the progression of AS and regulating lipid metabolism. In the context of AS, the specific effects of BBR on autophagy are not fully understood. Therefore, this study investigated the effect of BBR on autophagy in ApoE^−/−^ mice. In the ApoE^−/−^ + BBR group, the number of autophagic vacuoles and autophagosomal lysosomes was significantly higher than in the control and ApoE^−/−^ group (Figure 5A). The presence of LC3 in autophagosomes and its lipidation are known markers of autophagy (Wu et al., 2021). As shown in Figures 5B–E, the expression intensity of LC3B and the key autophagy protein Beclin-1 was significantly reduced in the ApoE^−/−^ group compared to the control group. After BBR intervention, the expression intensity of LC3B and Beclin-1 protein was significantly enhanced. In addition, P62 was selectively encapsulated into the autophagosome and degraded during autophagosome formation. The expression of P62 was significantly downregulated after BBR intervention (Figures 5D, E). These results suggest that BBR treatment can partially activate autophagy. Notably, BBR intervention further restored the expression level of autophagy-related proteins, suggesting a potential direct relationship between the anti-inflammatory effect of BBR and the activation of autophagy.

### 3.5 BBR reduces the degree of inflammation, activates autophagy, and repairs endothelial cells *in vitro*

**FIGURE 6:**
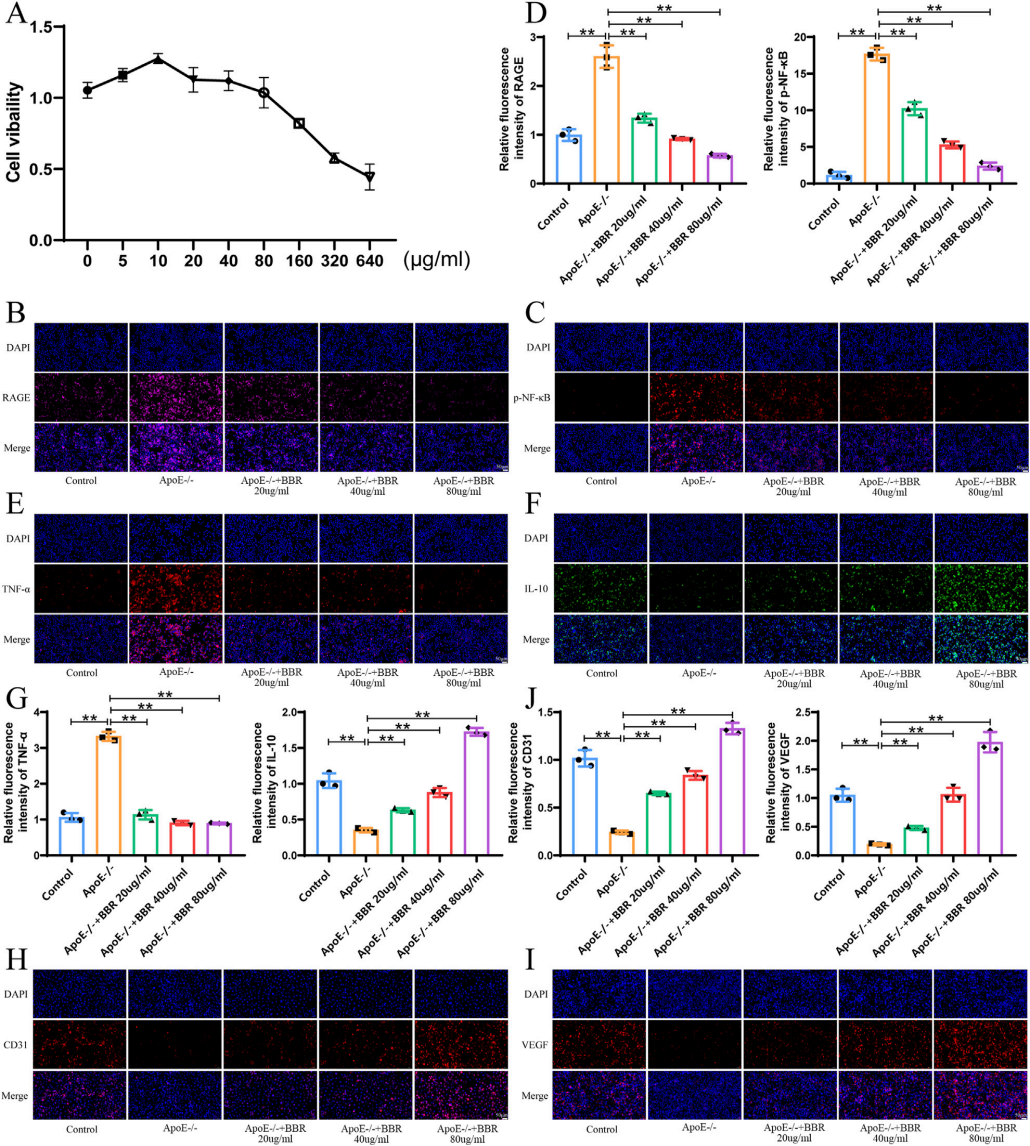
BBR reduces the degree of inflammation, activates autophagy, and repairs endothelial cells *in vitro*. **(A)** Cell viability of HUVEC cells incubated with BBR for 24 h was measured using CCK-8 assays (n = 3). **(B)** Immunofluorescence staining of RAGE after BBR treatment (Scale: 50 μm). **(C)** Immunofluorescence staining of p-NF-κB after BBR treatment (Scale: 50 μm). **(D)** Fluorescence staining quantitative analysis of RAGE and p-NF-Κb (n = 3). **(E)** Immunofluorescence staining of TNF-α in cells treated with BBR (Scale: 50 μm). **(F)** Immunofluorescence staining of IL-10 in cells treated with BBR (Scale: 50 μm). **(G)** Quantitative analysis of TNF-α and IL-10 by fluorescence staining (n = 3). **(H)** Immunofluorescence staining of CD31 in cells treated with BBR (Scale: 50 μm). **(I)** Immunofluorescence staining of VEGF after BBR treatment (Scale: 50 μm). **(J)** Quantitative analysis of CD31 and VEGF by fluorescence staining (n = 3).

Network pharmacology and *in vivo* experiments suggested that BBR may activate autophagy to alleviate inflammation through the modulation of proteins related to the RAGE-NF-κB signaling pathway. To further verify this hypothesis, we examined the expression levels of related proteins *in vitro*. Based on CCK-8 assay results, three concentrations of BBR (20, 40, and 80 μg/mL) were selected for *in vitro* experiments (Figure 6A). As shown in Figures 6B–D, the expression of RAGE and p-NF-κB was significantly downregulated in the ApoE^−/−^ + BBR group compared to the ApoE^−/−^ group, with the highest effect observed in the high-dose ApoE^−/−^ + BBR group. The expression of TNF-α was upregulated while IL-10 was downregulated in the ApoE^−/−^ group. However, BBR intervention significantly reversed these changes (Figures 6E–G). Furthermore, the high-dose ApoE^−/−^ + BBR group exhibited the most significant upregulation of endothelial cell markers CD31 and VEGF compared to the ApoE^−/−^ group (Figures 6H–J). These findings indicate that BBR effectively reduces inflammation and maintains vascular endothelial cell integrity *in vitro*.

### 3.6 Berberine elevates the autophagy in HUVEC cells

**FIGURE 7:**
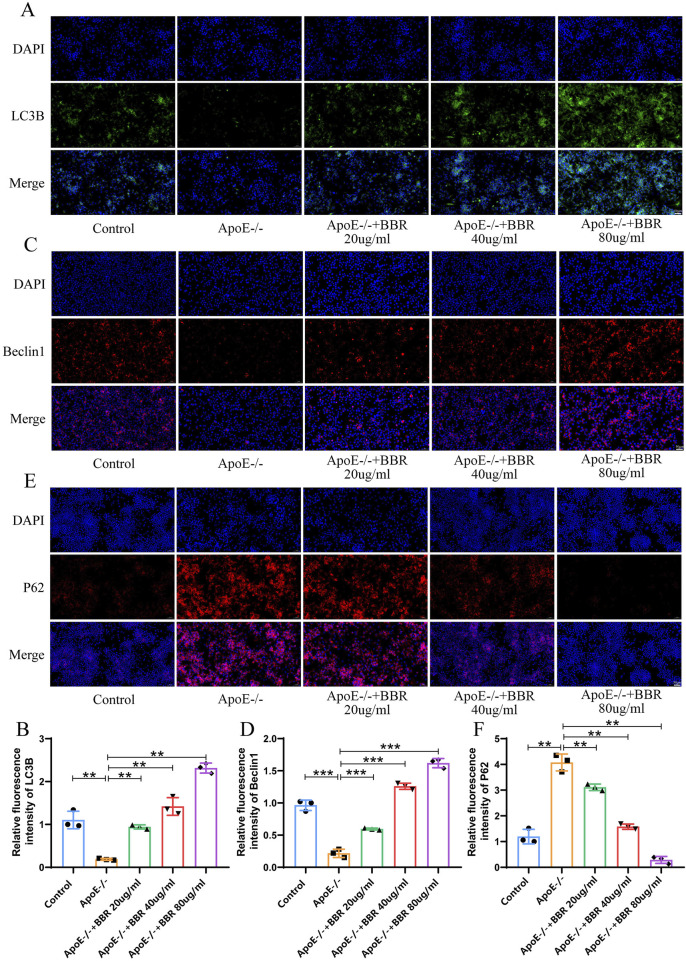
BBR elevates the autophagy level of HUVEC cells **(A)** Immunofluorescence staining of LC3B after BBR treatment (Scale: 50 μm). **(B)** Fluorescent staining quantitative analysis of LC3B (n = 3). **(C)** Immunofluorescence staining of Beclin1 after BBR treatment (Scale: 50 μm). **(D)** Fluorescence staining quantitative analysis of Beclin1 (n = 3). **(E)** Immunofluorescence staining of P62 after BBR treatment (Scale: 50 μm). **(F)** Quantitative analysis of fluorescent staining for P62 (n = 3).

Next, we investigated the impact of BBR on autophagy levels *in vitro*. Compared to the control group, autophagy level was significantly suppressed in the ApoE^−/−^ group, as indicated by increased P62 expression and significantly reduced LC3B and Beclin1 expression. Following BBR intervention, P62 expression was significantly downregulated, while LC3B and Beclin1 expression levels were significantly elevated, with the highest effects observed in the high-dose ApoE^−/−^ + BBR group (Figures 7A–F). These findings align with results from animal model experiments.

### 3.7 BBR promotes autophagy to alleviate inflammation via the RAGE-nf-κb signaling pathway

**FIGURE 8:**
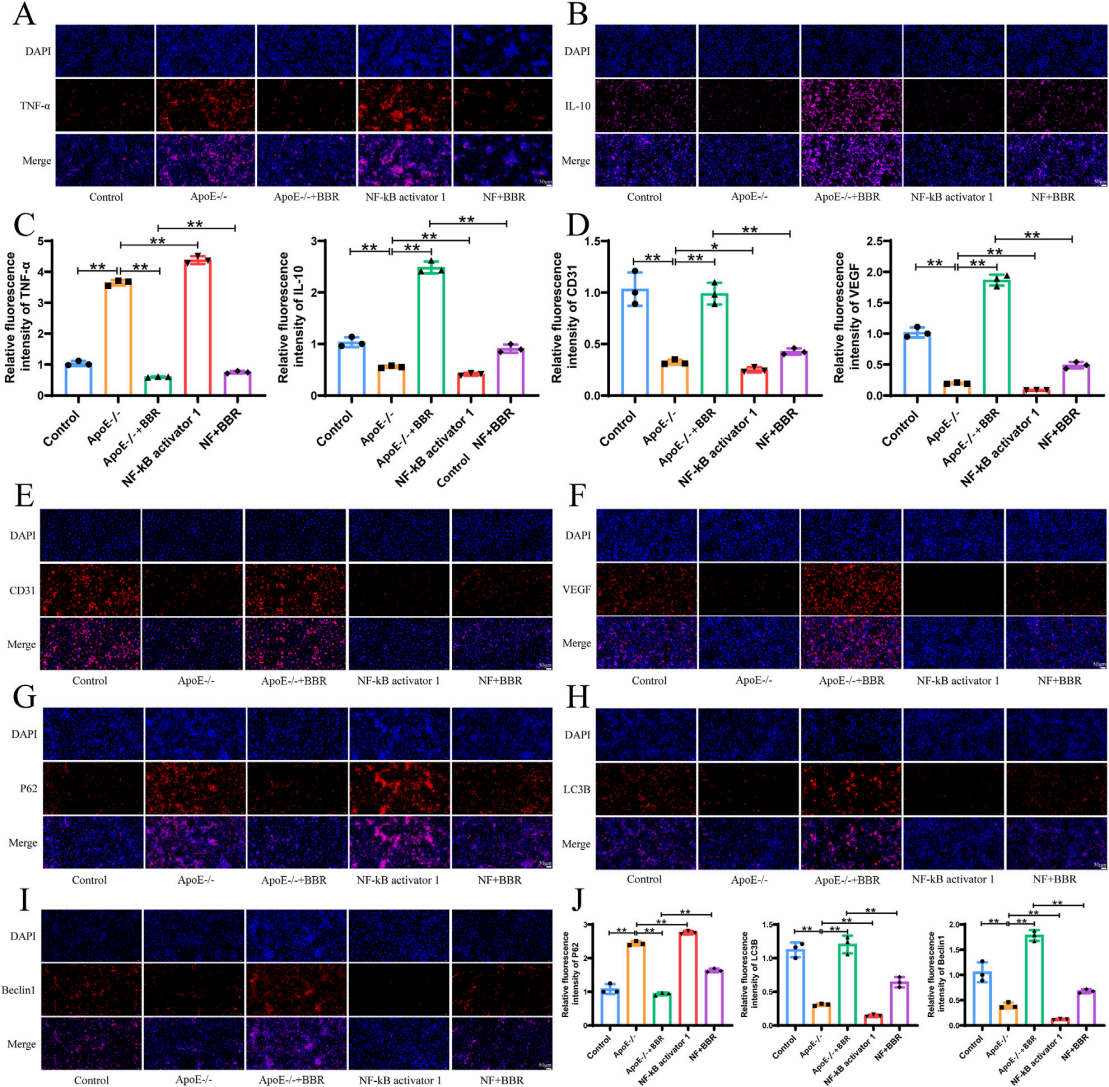
BBR promotes autophagy to alleviate inflammation via the RAGE-NF-κB signaling pathway. **(A, B)** Immunofluorescence of TNF-α and IL-10 in cell samples (Scale: 50 μm). **(C)** Quantitative fluorescence analysis of TNF-α and IL-10 (n = 3). **(D)** Quantitative fluorescence analysis of CD31 and VEGF. **(E–I)** Immunofluorescence of CD31, VEGF, P62, LC3B, and Beclin1 in cell samples (Scale: 50 μm). **(J)** Fluorescence quantitative analysis of CD31, VEGF, P62, LC3B, and Beclin1 (n = 3).

To explore the mechanism by which BBR regulates autophagy and inflammation, NF-κΒ activator 1 was employed for verification. Compared to the ApoE^−/−^ group, treatment with NF-κΒ activator 1 elevated the expression of TNF-α and reduced the expression of IL-10 (Figures 8A–C), suggesting that NF-κΒ activator 1 exacerbated the inflammatory response *in vitro*. Co-treatment with NF-κΒ activator 1 and BBR reversed the expression of TNF-α and IL-10 observed with the NF-κΒ activator alone, though to a lesser extent than with BBR treatment alone. BBR treatment significantly elevated the expression of CD31 and VEGF, preserving the integrity of vascular endothelial cells. Following NF-κΒ activator 1 intervention, CD31 and VEGF expression levels were markedly reduced compared to the ApoE^−/−^ group (Figures 8D–F), suggesting that an enhanced inflammatory response may compromise vascular endothelial cell integrity. Autophagy-related protein P62 expression was upregulated by NF-κΒ activator 1, while LC3B and Beclin1 expression decreased, consistent with the ApoE^−/−^ group. Co-treatment with NF-κΒ activator 1 and BBR significantly reversed the expression of P62, LC3B, and Beclin1 observed with the NF-κΒ activator alone (Figures 8G–J). These findings confirm that BBR activates autophagy through the RAGE-NF-κB pathway, reducing inflammation and repairing damaged vascular endothelial cells.

## 4 Discussion

At present, the exact mechanism underlying the development of AS is unknown. However, accumulating evidence suggests that autophagy plays a significant role in the occurrence and development of AS. BBR, which has a wide range of pharmacological and biological activities, has emerged as an over-the-counter (OTC) drug with a high oral bioavailability and minimal side effects. It has demonstrated beneficial effects across various conditions, including type II diabetes, polycystic ovary syndrome, metabolic syndrome, AS, congestive heart failure, hyperlipidemia, and certain cancers (Imenshahidi and Hosseinzadeh, 2019; Kheir et al., 2010). Studies have shown that BBR inhibits the development of AS and increases the stability of atherosclerotic plaques in ApoE−/− mice by activating ERK/JNK, AMPK, and PPARγ signaling pathways ((Wang et al., 2011; Li L. et al., 2016; Chen et al., 2014; Zhu et al., 2018). This study reveals a novel role for RAGE-mediated autophagy in the regulation of AS. The main findings are summarized as follows: Firstly, BBR intervention reduced aortic plaque deposition and facilitated the repair of damaged vascular endothelial cells. Secondly, BBR exerted its protective effects on AS progression by activating autophagy and reducing inflammatory responses. Finally, we discovered a significant association between RAGE-NF-κB axis-mediated autophagy and the protective effects of BBR.

Network pharmacology acts as a powerful tool for exploring the complex mechanisms underlying multi-metabolite TCM formulations (Zuo, et al., 2018). In this study, 62 cross targets of BBR, AS, lipid metabolism, and inflammation were related to the AGE-RAGE signaling pathway in diabetic complications, NF-κB signaling pathway, and neuroactive ligand-receptor interaction, suggesting that BBR might regulate inflammatory response. Based on the results of the enrichment analyses, further molecular docking experiments were conducted to explore the potential mechanism of BBR. The findings suggest that BBR shows a strong binding affinity for target proteins associated with the AGE-RAGE and NF-κB signaling pathways, indicating that BBR may slow down the progression of AS through the RAGE-NF-κB axis.

Autophagy is an important pathway in many biological processes and diseases and is essential for renewing and reusing cellular metabolites and energy homeostasis. The link between autophagy and diseases, including cancer (Ishaq, et al., 2020), neurodegenerative diseases (Mizushima et al., 2008), and atherosclerosis (Bravo-San Pedro et al., 2017), has attracted considerable attention. Studies have emphasized that, in the early stage of atherosclerotic lesions, autophagy activation is important for alleviating AS, while autophagy inhibition aggravates AS. The underlying mechanisms are complex. Autophagy activation has been shown to lead to a reduction in certain pro - inflammatory cytokines, such as monocyte chemoattractant protein - 1 (MCP - 1) and interleukin - 8 (IL - 8) (Sharmaet al., 2024). In our study, in accordance with the 4R rule (Reduce, Refine, Replace, Responsibility) and in combination with other animal experimental results (Wu et al., 2020), a dosage of 100 mg/kg body weight of BBR was administered by gavage once daily. BBR intervention increased the levels of autophagy-associated proteins LC3B and Beclin1 while decreasing P62 expression, indicating that BBR therapy can activate autophagy. Our findings from cultured cells were consistent with data from experiments in mice. In addition, we further investigated the mechanism by which BBR regulates autophagy using NF-κΒ activator 1. The results indicated that NF-κB activation suppressed autophagy, aggravated the inflammatory response, and undermined the integrity of vascular endothelial cells. These findings strengthen that BBR activates autophagy via the RAGE-NF-κB signaling pathway, thereby mitigating inflammatory responses associated with AS. The receptor for advanced glycation end products (RAGE) is a versatile cell surface molecule belonging to the immunoglobulin superfamily and is closely associated with diabetes, AS, Alzheimer’s disease, and vascular damage (Soro-Paavonen A,et al., 2008; Wang B, et al., 2017; Senatus L,et al., 2017; Paudel et al., 2020). RAGE expression is extremely low in normal cells but is elevated in chronic inflammation (Daffu G,et al., 2013). In this study, we found that intervention with BBR significantly downregulated the expression of RAGE. NF-κB, a key signaling pathway, regulates many inflammatory factors, including TNF-α, IL-1β, and IL-6 (Capece D,et al., 2022). Previous studies have shown that NF-κB inhibition can reduce foam cell formation (Kim and Kim, 2019). This study found that BBR intervention downregulated the expression of NF-κB and IL-10 while upregulating the expression of TNF-α. This regulation alleviated inflammation and repaired vascular endothelial cells. Notably, the anti-inflammatory effect of BBR was weakened with the addition of NF-κB activators, suggesting that BBR mitigates inflammation and activates autophagy through the NF-κB pathway.

## 5 Conclusion

In summary, this study suggests that BBR can mitigate AS by inhibiting inflammation, primarily by enhancing autophagy through the RAGE-NF-κB signaling pathway. These findings suggest that BBR may serve as a potential candidate for AS treatment, warranting further clinical investigation.

## Data Availability

The original contributions presented in the study are included in the article/Supplementary Material, further inquiries can be directed to the corresponding author.

## Supplementary material

SUPPLEMENTARY FIGURE 1 3D and 2D visualizations of BBR docking with ICDK4, F3, ICAM1, JAK2, MAPK10, MAPK14, PIM1, RAC1, and PTGS2.

## Abbreviations

AS, atherosclerosis; BBR, berberine; TC, total cholesterol; TG, triglyceride; LDL-C, low-density lipoprotein cholesterol; HDL-C, high-density lipoprotein; HE, hematoxylin and eosin; HFD, high-fat diet; ox-LDL, low-density lipoprotein; LDL-C, low-density lipoprotein cholesterol; HDL-C, high-density lipoprotein cholesterol.
