# Supplementary material for: Berberine alleviates atherosclerosis by modulating autophagy and inflammation through the RAGE-NF-κB pathway
Source: Front Pharmacol. 2025 Mar 31;16:1540835. doi: 10.3389/fphar.2025.1540835 (PMC11994719; doi:10.3389/fphar.2025.1540835)
Supplement: Supplementary file 1 [file Table1.docx]

Supplementary Table 1

| **Core active molecule** | **S value** | | | | | | | | | | | |
| --- | --- | --- | --- | --- | --- | --- | --- | --- | --- | --- | --- | --- |
|  | **CDC42** | **CDK4** | **MAPK14** | **F3** | **ICAM1** | **JAK2** | **PIM1** | **MAPK10** | **RAC1** | **IKBKB** | **LCK** | **PTGS2** |
| **Berberine** | -5.9317 | -6.5752 | -6.2431 | -5.8413 | -6.1179 | -5.7886 | -5.8317 | -6.7239 | -6.1874 | -5.1801 | -6.0971 | -5.9395 |
| **Pathway** | AGE-RAGE | | | | | | | | | NF-κB | | |
