# Supplementary figures and images for: Berberine alleviates atherosclerosis by modulating autophagy and inflammation through the RAGE-NF-κB pathway
Source: Front Pharmacol. 2025 Mar 31;16:1540835. doi: 10.3389/fphar.2025.1540835 (PMC11994719; doi:10.3389/fphar.2025.1540835)

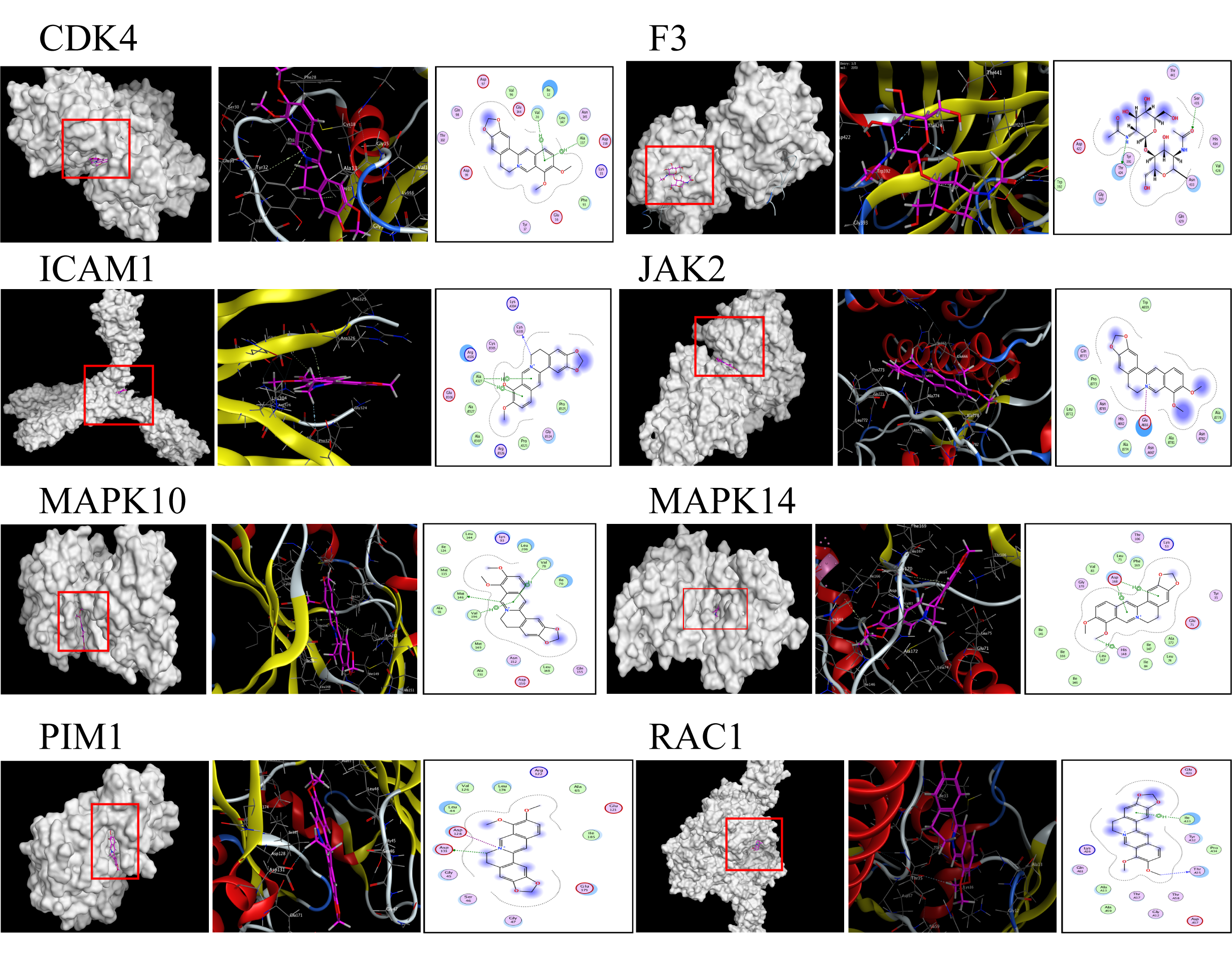

Supplement: Supplementary file 2 [file Image1.tif]
